# Parental Response to Only Children: Breaking the Stereotypes

**DOI:** 10.3390/children8070605

**Published:** 2021-07-17

**Authors:** Rona L. Levy, Tasha B. Murphy, Kendra Kamp, Shelby L. Langer, Miranda A. L. van Tilburg

**Affiliations:** 1 School of Social Work, University of Washington, Seattle, WA 98105, USA; tbmurphy@uw.edu (T.B.M.); vantilburg@campbell.edu (M.A.L.v.T.); 2 School of Nursing, University of Washington, Seattle, WA 98195, USA; kamp@uw.edu; 3 Edson College of Nursing and Health Innovation, Arizona State University, Phoenix, AZ 85004, USA; shelby.langer@asu.edu; 4 College of Pharmacy & Health Sciences, Campbell University, Buies Creek, NC 27506, USA; 5 School of Medicine, University of North Carolina, Chapel Hill, NC 27599, USA

**Keywords:** parenting, parental factors, birth order, school absences, social learning, illness behavior, protectiveness

## Abstract

While much has been written about the relationship between only child status and parents’ behavior toward children, and consequent personality and intelligence, little is known about the relationship between only child status, parental response to illness, and subsequent child illness behavior. In this study, 227 mothers of 342 children completed measures designed to assess: (a) their children’s school attendance, (b) their own psychological status, and (c) their own responses to their children’s expressions of stomach pain. Parents of only children were more likely to minimize their children’s gastrointestinal symptoms than were parents of children with at least one sibling. In addition, only children were less likely to miss school. Parental protectiveness did not differ as a function of only child status. These findings are somewhat discrepant with commonly held beliefs about parents’ patterns of responding to only children.

## 1. Introduction

Much has been written about the effects of birth order, including only child status, on parents’ behavior toward children and consequent personality, intelligence, and behavior development [1,2,3,4]. Certainly popular characterizations of parents’ behaviors of only children have presented them as coddled or spoiled, receiving excessive amounts of attention, resulting in adverse traits. One very early 19th century researcher from Clark University in Massachusetts [5] reported that nearly all of his 200 survey respondents, when asked about only children they knew, described children without siblings as excessively spoiled. This perception continued, with the belief during the 20th century that an only “child would become overly sensitive and eventually a hypochondriac with weak nerves” [6].

Research in these areas has often found, in many cases contrary to popular beliefs, that the only child status has not been associated with poor outcomes in several areas [7,8,9,10,11]. For instance, a commonly held stereotype was that only children were more likely to be narcissistic; however, research indicates that only children have similar levels of narcissism as individuals with siblings [9,10]. Other research in both Western and non-Westernized countries has indicated that birth order has little impact on intelligence, personality, and risk aversion [12,13,14]. Studies which report significant associations between birth order and personality often have very small correlations (r = 0.02), leading authors to report the impact of birth order on personality is negligible [3].

With escalation in health care costs, among other factors, one important area which has received recent attention, but has been limited in the birth order literature, is the investigation of the phenomenon commonly called “illness behavior.” Illness behavior is a term that refers to the way people perceive and react to somatic sensations (i.e., bodily sensations) which might signify disease [15]. Illness behavior falls on a continuum, ranging from failure to notice such sensations or refusal to let even serious illness interrupt one’s normal routine, to misinterpretation of normal somatic sensations as symptoms of disease and seeking unnecessary medical treatment for minor complaints. Both ends of the continuum are of concern to health care providers: denial may lead individuals to postpone diagnostic investigations or to not adhere to medical regimens. Preoccupation with illness, on the other hand, creates a costly burden on the health care system [16,17].

Social scientists have been exploring whether illness behavior might be learned. For example, a child who grows up in a family with an ill parent may observe the parent’s maladaptive illness behaviors (i.e., complaints, activity avoidance) and then emulate these behaviors themselves. Some support for this has come from findings that one form of illness behavior, health care utilization, tends to run in families. Lewis and colleagues [18] found that children’s utilization of pediatric services was predicted by their mothers’ healthcare utilization. Schor et al. [19] found, in 693 families enrolled in a prepaid health plan, significant correlations in uses of services among family members, especially at the extremes of usage. The influence of parental healthcare utilization on children’s health care utilization for acute, chronic, and non-illness settings was 2.5 times higher for mothers compared to fathers. These familial utilization rates were correlated over six years of follow-up.

The concept of intergenerational transmission of illness behaviors was further explored by Levy and colleagues [20] and van Tilburg and colleagues [21] in studies of children of parents with Irritable Bowel Syndrome (IBS), a gastrointestinal complex of symptoms including chronic pain with no known etiology and high rates of disability [22,23,24,25]. They found children whose parents had a diagnosis of IBS had significantly higher healthcare utilization rates, not just for gastrointestinal symptoms but also for a wide range of somatic symptoms, as compared to a control group of children whose parents were not diagnosed with IBS. It is perhaps not surprising, then, that a recent systematic review of 27 studies of children with a chronically ill parent concluded that parental illness is related to increased somatic symptoms in children [26]. The relationship between increased somatic symptoms in children with chronically ill siblings was mixed, further highlighting the concept of intergenerational transmission [26].

Further research has explored the mechanisms by which this learning might occur. Using retrospective data, Whitehead et al. [27] found that subjects with IBS were more likely to report that when they had a cold as a child, their parents gave them toys, gifts, or treat foods such as ice cream, and those who received these rewards as children endorsed significantly higher levels of illness behavior. Using prospective data, Levy and colleagues [28] found that children whose mothers made protective responses to illness complaints independently reported more severe stomachaches and also had more school absences for stomachaches. In these studies and others [29,30,31], parental reinforcement of illness symptoms resulted in increased symptom complaints and doctor visits and reduced school attendance. Thus, child illness behaviors are associated with parental responses; however, what has rarely been studied is whether there are differences in child illness behaviors and parental response based on whether a child has siblings.

One early study found that mothers of pre-term and sick infants were more responsive to first-borns than later-borns such that first-born preterm and sick infants had more maternal simulation and responsiveness compared to later-born pre-term and sick infants [32]. Another study found that birth order was directly associated with the frequency of use of general practitioners [33]. Richards and Goodman [8] found that parents of only children in a psychiatric clinic differed from other parents in that they were more willing or able to seek medical advice. Similarly, Kushnir [34] concluded in a study of emergency room visits and birth order, that parental over-concern and protectiveness may predispose firstborns to perceive illness and painful situations as more stressful. In another study, Hawken [35] concluded that heightened parental concern was related to first-born children having a higher incidence of emergency room visits and hospitalizations following vaccinations. However, these studies were not designed to explore the specific ways these environmental mechanisms operate to shape these traits.

The current study sought to investigate whether parents respond differently to children’s symptoms as a function of the children’s only child status. Based on prior findings of greater parental concern for only children [1,11,36,37], we hypothesized that parents would show greater protectiveness to these children as compared to children with at least one sibling. Furthermore, based on our own research cited above into the effects of protectiveness, we also hypothesized that increased protectiveness would result in greater illness behavior, in the form of school absenteeism, on the part of these children.

## 2. Materials and Methods

### 2.1. Participants

The present report focuses on a subsample from a larger study that examined illness behavior and parental response of mothers both with a diagnosis of IBS (“Case” parents) and without a diagnosis of IBS (“Control” parents), a chronic gastrointestinal pain disorder [28]. In the parent study, IBS (case) families were randomly selected from an automated patient database at a large health maintenance organization (Group Health Cooperative in Seattle, WA, USA) to identify women diagnosed with IBS or abdominal pain during the preceding two years and who had 1 or more children between the ages of 8 and 15. Trained telephone interviewers conducted further screening to ensure the mother met the Rome criteria for IBS (the Rome criteria are the current worldwide accepted symptom-based diagnostic criteria for IBS) [38]. Control families were selected from the database by identifying women who did not have a clinic visit during the preceding 2 years for IBS, abdominal pain, constipation, or diarrhea. Controls were further screened by telephone interview to ensure that neither the mother nor any adult family member met the Rome criteria for IBS. Cases were matched to controls with respect to age and number of children. Other inclusion criteria for both cases and controls included: the mother needed to be the child’s legal guardian, and the child needed to have lived with the parent at least half of the time in the previous 2 years, presence of disability in the eligible child requiring full time special education, and no other family member had a diagnosis of ulcerative colitis or Crohn’s disease. The subsample for the present study was derived by including only mothers who completed the questionnaires for all children in the family. This resulted in a subsample of 227 mothers of 342 children.

Study procedures were approved by the Institutional Review Board of Group Health Cooperative. All participants provided informed consent/assent.

### 2.2. Measures

Mothers completed pencil-and-paper questionnaires either in the clinic or in their home (their choice). Measures are described below.

School absences, doctor visits, and medication use. As a measure of illness behavior, mothers were asked to report the number of days during the past 3 months that their child missed school, the number of times the child visited a doctor, and the frequency of medication use as a result of abdominal pain. The response options for school absences and doctor visits were none, 1–3, 4–6, 7–9, or 10+. Response options for medication use were never, every month, every week, more than once a week, and every day. These variables were dichotomized as none (0) or 1 (one or more days). The decision to do so was based on the distribution of the responses.

Adult Responses to Children’s Symptoms (ARCS) [39]. This scale, an extension of the Illness Behavior Encouragement Scale [40], assesses parental responses to child abdominal pain symptoms. Factor-analytically derived subscales include protectiveness (13 items) and minimization (7 items). Protectiveness measures parental encouragement of child illness behavior, putting the child in a sick role. Minimization measures parental criticism of child illness behavior, encouraging the maintenance of normal responsibilities. Exemplar items for protectiveness are: “When your child has a stomachache or abdominal pain, how often do you… bring your child special treats or little gifts, …let your child stay home from school, or…tell others in the family not to bother your child or to be especially nice?” Exemplar items for minimization are: “When your child has a stomachache or abdominal pain, how often do you… tell your child not to make such a fuss about it, …try not to pay attention to your child, or …insist that your child try to go to school?” Ratings are made on a 0–4 (never to always) scale. Mean scores for each subscale are reported, with higher values indicative of greater protectiveness or minimization (theoretical range = 0–4).

With respect to the psychometric properties of the scale, the developers report internal consistencies (Cronbach coefficient α) of 0.87 for protectiveness and 0.67 for minimization [39]. Predictably, the two scales correlate inversely, r = −0.22, *p* < 0.01). Validity of the protectiveness subscale is supported by its relationship to health care utilization for gastrointestinal symptoms [41].

The Symptom Checklist 90-R [42] measures global distress. We focused here on the depression (13 items), anxiety (10 items), and somatization (12 items) subscales. Respondents were asked to rate how much they were bothered by various symptoms during the prior seven days, including “headaches”, “nervousness or shakiness inside”, “the idea that something serious is wrong with your body”, and “nausea or upset stomach”. Items were rated with respect to their bothersomeness over the past 7 days, using a 0 (not at all) to 4 (extremely) scale. The developers report strong reliabilities. Internal consistencies (Cronbach coefficient alpha) range from 0.85 to 0.90. Test–retest reliabilities range from 0.68 to 0.86. Validity is also strong, as evidenced by appropriate relationships with other measures of the constructs, e.g., the Middlesex Hospital Questionnaire.

### 2.3. Data Analysis

Analyses were conducted using SPSS statistical software. Demographic and clinical characteristics for mothers and children were described using percentages and numbers for categorical data and mean and standard deviations for continuous variables. Unpaired t-tests were used to examine maternal protectiveness and minimization, and SCL-90 depression, anxiety, and somatization as a function of only child status (only child versus child with at least one sibling). Cross-tabulation and chi square analyses were used to examine school absences, doctor visits, and medication use as a function of only child status. A *p*-value of 0.05 was considered statistically significant.

## 3. Results

### 3.1. Sample Demographics

Table 1 and Table 2 display the demographic and clinical characteristics of the sample. Children were, on average, 12 years old, 51% female, and 80% Caucasian. Forty percent were only children; 27%, first born; 27%, second born; 6%, third born or higher. Fifty-four percent had experienced some degree of abdominal pain in the past two weeks. Mothers of only children were, on average, 44 years old, and mothers of multiple children were, on average, 43 years old. Similar to children, mothers of both only children and multiple children were predominantly (more than 80%) Caucasian. Approximately half of the mothers (of both only children and multiple children) were college-educated, and more than 80% were employed either full time or part time. Additionally, 51% of mothers of only children had received a diagnosis of Irritable Bowel Syndrome compared to 46% of mothers of multiple children. Chi-square analysis found no significant differences between mothers of only children and mothers of multiple children for these demographic variables. Maternal living situation, however, differed between mothers of only children and mothers of multiple children; a greater percentage of mothers of only children were single mothers and had other adults living in the home compared to mothers of multiple children (χ^2^ = 8.190 (*p* < 0.05)).

### 3.2. Maternal Responses to Illness Behavior

Table 3 shows the results from the ARCS and SCL-90 questionnaires. While scores on the ARCS protectiveness subscale were slightly lower for mothers of only children (1.71 vs. 1.79), these differences were not significant. Maternal minimization was higher for mothers of only children (1.13 vs. 0.98); while these group differences approached significance, the effect size was not neligible.

### 3.3. Maternal Psychological State

Table 3 also displays the results for SCL-90 depression, anxiety, and somatization. Mothers of only children reported significantly higher levels of depression (0.85 vs. 0.64, *p* = 0.02) and anxiety (0.61 vs. 0.40, *p* = 0.007) compared to mothers of multiple children, but there were no significant differences for somatization (0.86 vs. 0.70, *p* = 0.061).

There were no differences in SCL-90 somatization scores by maternal race, marital status, number of children, child sex, or maternal age. There was a small, but significant correlation between SCL somatization scores and child age (r = 0.16, *p* < 0.05). Maternal education was associated with SCL-90 somatization. Mothers with a college education of less than 4 years reported higher somatization scores than mothers with a college degree (see Table 4). Maternal employment status (full-time vs. unemployed or part-time employment) was also associated with SCL-90 somatization. Full-time working mothers reported significantly higher somatization compared to unemployed/part-time working mothers (Table 4). Maternal IBS status was also associated with SCL-90 somatization: mothers with IBS reported significantly higher somatization than mothers without IBS (Table 4).

There were no differences in SCL-90 anxiety scores by maternal race, education, marital status, employment, age, or child sex. Child age was not significantly correlated with anxiety scores. Maternal IBS status, however, was associated with SCL-anxiety. Mothers with IBS reported significantly higher anxiety than non-IBS mothers (Table 4). Mothers of only children also reported significantly higher anxiety than mothers of multiple children (Table 4).

There were no differences in SCL-90 depression scores by maternal race, marital status, employment status, age, or child sex. Child age was not significantly correlated with depression scores. Maternal education was associated with SCL-90 depression scores: mothers with a college degree reported significantly less depression than mothers with a less than college education (Table 4). Maternal IBS status was also associated with SCL-90 depression scores: mothers with IBS reported significantly higher depression scores than mothers without IBS (Table 4). Number of children was also associated with depression scores, with mothers of only children reporting significantly higher depression scores than mothers of multiple children (Table 4).

Standardized residuals were analyzed to identify any outliers, which showed that one participant needed to be removed for Somatization, four participants needed to be removed for Anxiety, and seven needed to be removed for Depression. Multicollinearity was not a concern with these data (Tolerance ranged from 0.85 to 0.97 for Somatization, from 0.81 to 0.96 for Anxiety, and from 0.91 to 0.96 for Depression, and VIF ranged from 1.02 to 1.17 for Somatization, from 1.04 to 1.232 for Anxiety, and from 1.04 to 1.23 for Depression). The data also met the assumption of independent errors (Durbin–Watson value = 1.84 for Somatization, 1.98 for Anxiety, and 1.75 for Depression). Evaluation of the histogram and P-Plot of standardized residuals for all regressions indicated that the data were normally distributed. Finally, the scatterplot of standardized predicted values showed that the data met the assumptions of homogeneity of variance and linearity for all regressions (Appendix A).

Using the enter method, we found that the demographic variables in the model explained a significant amount of the variance in SCL-90 Somatization (F(9208) = 10.44, *p* < 0.001, R^2^ = 0.31, R^2^_Adjusted_ = 0.28), Anxiety (F(9205) = 4.32, *p* < 0.001, R^2^ = 0.16, R^2^_Adjusted_ = 0.12), and Depression (F(9203) = 7.52, *p* < 0.001, R^2^ = 0.25, R^2^_Adjusted_ = 0.22). The analysis showed that maternal IBS status and number of children significantly predicted somatization, anxiety, and depression (see Table 5). Child age also predicted somatization.

### 3.4. School Absences, Health Care Visits, and Medication Use

School absences, healthcare visits, and medication use were analyzed for the full sample of 342 children, as these variables are child-specific. Overall, 72.5% percent of children represented in the sample missed no days of school, 24.3% missed 1–3 days, and 4% missed 4 or more days. Similarly, 90.1% of children did not visit a doctor (8.5% had one visit, 1.5% had two or more visits), and 72.7% did not use medication during the prior 3 months (23.3% used medication every month, and 3.9% used medication at least once per week).

A cross-tabulation was used to examine the number of school days missed in the past three months due to stomachaches or abdominal pain (coded 0 for none and 1 for one or more) as a function of only child status. Children with siblings were more likely to miss school as compared to only children (33.2% versus 19.0%; see Table 6 below).

In addition to school days missed, mothers reported on the number of days their child visited the doctor for stomachaches/abdominal pain (coded 0 for no visits and 1 for one or more visits) and the frequency of medication use for stomachaches or abdominal pain (coded 1 for took no medications and 1 for took medications at least once/month) during the past 3 months. Regarding doctor visits, 10.2% of only children and 9.8% of children with siblings visited the doctor within the prior 3 months. Regarding medication use, 28.8% of only children and 26.3% of children with siblings took medicine for stomachache or abdominal pain within the prior 3 months. A cross-tabulation comparing only children and children with siblings was not significant for either doctor visits or medication use (Table 6).

## 4. Discussion

Prior studies in the literature have shown that parental protectiveness in response to illness behavior is related to increased symptoms and disability. We hypothesized that parents of only children would exhibit greater protectiveness than parents of multiple children. The present study, however, did not find parents more protective in response to only children’s symptom complaints. We also found no differences in doctor visits or medication use (which might be seen as protective behaviors) between only children and children with siblings. These findings are somewhat discrepant with commonly held beliefs that parents tend to be over-protective toward only children. They are also inconsistent with Richards and Goodman’s [8] finding of parents of only children being more willing or able to seek medical advice. However, their study only found increased health care seeking by parents of only children, not the specific form of responses we were investigating. Arguably, these day-to-day interaction behaviors may have a more significant impact on personality than doctor visits. In addition, in the Richards and Goodman study, health care seeking referred to psychiatric care and thus may not generalize to other medical situations.

Overprotective parenting includes doing tasks for children that they developmentally should be able to do themselves, as well as removing barriers and potential harms that children developmentally can manage themselves. Overprotective parenting has been associated with a host of negative outcomes for parents and children. For example, parental overprotectiveness has been associated with child anxiety, behavior disorders, suicidality, adolescent alcohol use, and increased child pain behaviors [43,44,45,46,47]. Yet, it has universal appeal in society today: overprotective parenting is often agreed upon as ‘good parenting’ by people across socioeconomic classes [48]. This may explain why protectiveness would not differ between parents with an only child and parents with multiple children. Overprotective parenting is culturally expected and therefore applied equally to all children.

We did not find differences in protectiveness between parents of only children and parents of multiple children. While we also did not find differences in parental minimization of child symptoms between parents of only children and parents of multiple children, this comparison approached significance (*p* = 0.55), such that minimization scores were higher among parents of only children than parents of children with at least one sibling. This suggests that parents of only children may be more likely to discount, criticize, or ignore their child’s pain complaints and behaviors and send their children to school even when they are experiencing pain or illness. In fact, our study found that children with siblings were more likely to miss school. While it could be argued that children in multiple families may be more likely to become ill due to greater illness exposure, it is intriguing to speculate that the phenomenon we have uncovered (more parental minimization in response to only children’s expressions of symptoms) may also contribute to this attendance behavior. It should also be noted that although parents were clearly electing to keep children with siblings home from school more frequently for abdominal symptoms, they were not giving their children more medications or taking them to the doctor more frequently. Speculation as to reasons for this phenomenon could go in many directions. For example, it could be suggested that parents of multiple children might evaluate symptoms of any one child in comparison to symptoms of their other children.

Finally, the fact that maternal psychological distress differed as a function of only child status is also intriguing. In our study, mothers of only children reported higher somatization, anxiety, and depression than mothers of multiple children, and the number of children was shown to be a significant predictor even after controlling for various demographic factors. It is not clear that this emotional distress is due to the child’s status. It could be argued that mother’s distress may have prevented them from having more children. Our analyses showed that the only child status alone, however, did not fully explain the somatization, anxiety, and depression scores. Mothers with IBS reported higher scores on all SCL-90 subscales compared to mothers without IBS. Maternal IBS status (i.e., having an IBS diagnosis) was also a significant predictor of somatization, anxiety, and depression. This makes intuitive sense, as mothers who themselves have an IBS diagnosis may suffer pain and other symptoms. One might expect that mothers with less than a college education, with multiple children, and single parenthood would experience the greatest emotional distress, but this was not our finding. While our t-test analyses initially indicated that mothers with less than a college education reported higher somatization and depression scores than college-educated mothers, maternal education was not a significant predictor in the regression analyses, which controlled for the effect of other variables. In a recent, non-peer reviewed study, maternal stress was higher in moms who had two or three children than in mothers of single children [49]. High parental stress can have negative outcomes for children, including health-related outcomes. For example, in a national US study, parent stress increased the likelihood for the child to present at pediatric emergency care [50]. During the 2020 pandemic, adults with children reported higher levels of stress than adults without children [51]. Parents also reported high levels of physical symptoms related to stress such as stomachaches, headaches, eating and sleeping problems [52]. When parents are sick, they model to their children how to respond to symptoms, which influences child illness behaviors and health outcomes [53,54,55]. This may suggest that any effect of only child status on child health outcomes may be mediated by parental distress, parental symptoms, and parental illness behaviors. There could also be other psychological factors which may be associated with a choice to have only one child. This needs to be examined in future studies.

One limitation of this study lies in the self-report nature of the measures. Future research should endeavor to observationally measure parental responses to child symptom complaints and link these behaviors to the only child status. Additionally, this study focused on self-identified primary caregivers, and in this study those who identified as such were mothers. It would be interesting to examine the behaviors of fathers when their children complain of illness in only child situations. Furthermore, parent behaviors may change with time. For example, after birth, parent behaviors to their first born change in half of the parents, mostly negative [56]. These negative behaviors are a function of feeling exhausted and will likely change as the newborn matures.

Despite these considerations, this study represents an important methodological and conceptual shift in the literature examining the relationship between only child status and parental response to only children. Methodologically, this study examines *specific* child behaviors and *specific* parental behavioral responses of only children and their parents. Thus, it goes beyond more global examinations of traits of only children. Conceptually, this study also looks at these behaviors and responses in an area new to the only child literature—the development of illness symptoms and, ultimately, illness behavior and the parental factors that may, or may not, contribute toward this development. For instance, when a child reports a physiological experience to a parent, the parent may or may not respond. And *if* the parent responds, *how* they respond may set in place a process which can have a lasting effect on that child’s symptom experience, quality of life, and disability. For example, a parent may, with their reaction, communicate “This is nothing to pay attention to”, or “This is serious, and you should notice it whenever it occurs”, and/or “You definitely should complain about this”, and/or “You should curtail normal activities as a result of this”, etc. All of these responses, with the exception of the first response, are likely to increase the likelihood that the child will notice the sensation in the future and react to it in a way that limits their regular daily events. Because the encouragement of illness behavior in childhood can also lead to high levels of inappropriate illness behavior in adulthood [18], the findings here may have implications not only for children, but also for these individuals in later life. Further research on specific parental behaviors which could contribute to other behaviors and traits of only children is one logical and productive future direction for this work.

## Figures and Tables

**Table 1:** Demographic and clinical characteristics of the children (*n* = 342).

Age	Mean = 12.03 (SD = 2.46)
Gender(male)	*n* = 167 (48.8%)
Race/ethnicity, *n* (%)	
Asian	16 (4.7%)
African American	19 (5.6%)
Hispanic	16 (4.7%)
Native American	3 (0.9%)
Caucasian	274 (80.1%)
Other	11 (3.2%)
Unknown	3 (0.9%)
Birth order, *n* (%)	
Only child	137 (40.1%)
Child with 1+ sibling	205 (59.9%)

**Table 2:** Demographic and clinical characteristics of the mothers (*n* = 227).

	Mothers of Only Children	Mothers of Multiple Children
	(*n* = 135)	(*n* = 92)
Age	Mean = 44.35 (SD = 7.43)	Mean = 42.55 (SD = 5.14)
Race/ethnicity, *n* (%)		
Asian	6 (4.4%)	4 (4.3%)
African American	9 (6.7%)	3 (3.3%)
Hispanic	4 (3.0%)	5 (5.4%)
Native American	1 (0.7%)	1 (1.1%)
Caucasian	109 (80.7%)	75 (81.5%)
Other	5 (3.7%)	3 (3.3%)
Unknown	1 (0.7%)	1 (1.1%)
Educational status, *n* (%)		
<High school	4 (2.9%)	0 (0.0%)
High school degree	6 (4.4%)	13 (14.1%)
Some college or		
Technical school	53 (39.3%)	34 (37.0%)
4. -year college	31 (23.0%)	18 (19.6%)
>College/Graduate	40 (29.6%)	27 (29.3%)
School		
Unknown	1 (0.7%)	0 (0.0%)
Employment status, *n* (%)		
Unemployed	19 (14.1%)	16 (17.4%)
Employed part-time	22 (16.3%)	29 (31.5%)
Employed full-time	92 (68.1%)	46 (50.0%)
Unknown	2 (1.5%)	1 (1.1%)
Living situation, *n* (%)		
Single mom	33 (24.4%)	11 (12.0%)
Other adult in home	15 (11.1%)	7 (7.7%)
Married, spouse	84 (62.2%)	73 (79.3%)
In home		
Separated	2 (1.5%)	1 (1.1%)
Unknown	1 (0.7%)	0 (0.0%)
Maternal IBS status, *n* (%)		
IBS case	69 (51.1%)	42 (45.7%)
Control	66 (48.9%)	50 (54.3%)

**Table 3:** Maternal protectiveness, minimization, and distress as a function of only child status.

	Only Child Mean (SD)	Child with Siblings Mean (SD)	*t*	*p*	Effect Size *
ARCS protectiveness	1.71 (0.71)	1.78 (0.64)	−0.81	0.420	0.10
ARCS minimization	1.13 (0.60)	0.98 (0.51)	1.93	0.055	0.27
SCL-90 anxiety	0.61 (0.67)	0.40 (0.47)	2.72	0.007	0.36
SCL-90 depression	0.85 (0.71)	0.64 (0.59)	2.33	0.021	0.32
SCL-90 somatization	0.86 (0.72)	0.70 (0.56)	1.88	0.061	0.25

* Cohen’s d.

**Table 4:** Significant *t*-test findings by demographics on SCL-90 subscales.

	Mean (SD)	*t*	*p*	Effect Size *
Somatization Subscale				
Maternal education		2.11	0.04	0.27
<4 year college	0.89 (0.70)			
College degree or higher	0.71 (0.61)			
Maternal employment status		−2.3	0.02	0.30
Full time	0.87 (0.72)			
Unemployed or part-time	0.68 (0.55)			
Maternal IBS status		−8.66	0	
IBS diagnosis	1.14 (0.70)			
No IBS diagnosis	0.47 (0.42)			
Anxiety Subscale				
Maternal IBS status		−4.39	0	0.58
IBS diagnosis	0.70 (0.66)			
No IBS diagnosis	0.36 (0.49)			
Number of children		2.72	0.007	0.36
Only child	0.61 (0.67)			
Multiple children	0.40 (0.47)			
Depression Subscale				
Maternal education		2.14	0.04	0.29
<4 year college	0.86 (0.73)			
College degree or higher	0.67 (0.59)			
Maternal IBS status		−5.68	0	0.76
IBS diagnosis	1.01 (0.67)			
No IBS diagnosis	0.54 (0.59)			
Number of children		2.4	0.02	0.32
Only child	0.85 (0.71)			
Multiple children	0.64 (0.59)			

* Cohen’s d multiple regression was used to determine the relative effect of the demographic variables. All demographic variables (maternal race, education, marital status, employment status, IBS status, number of children, child age, and child sex) were included in the regressions as predictor variables for each of the SCL-90 subscale scores. Maternal age, ethnicity, education, employment status, marital status, and child sex were used in the regression model as dichotomous variables (maternal age ≤43 or 44+ based on median split; ethnicity = Caucasian or not Caucasian; education ≤college or 4-year college degree or higher; marital status = married or not married; employment = full time or part time/unemployed).

**Table 5:** Multiple regression predicting SCL-90 scores.

SCL-90 Subscale	Predictor	*β*	T	*p*
Somatization	Maternal IBS Status	0.48	8.04	<0.001
	Child Age	0.18	2.94	<0.01
	Child sex	0	−0.01	ns
	Maternal Age	0.01	0.2	ns
	Maternal Ethnicity	−0.08	−1.41	ns
	Maternal Education	−0.01	−0.09	ns
	Marital status	0.01	0.04	ns
	Employment status	0.05	0.83	ns
	Number of children	−0.13	−2.19	<0.05
Anxiety	Maternal IBS Status	0.33	4.96	<0.001
	Child Age	0.09	1.25	ns
	Child sex	0.03	0.44	ns
	Maternal Age	−0.01	−0.08	ns
	Maternal Ethnicity	−0.05	−0.7	ns
	Maternal Education	0.01	0.16	ns
	Marital status	0.06	0.84	ns
	Employment status	−0.02	−0.35	ns
	Number of children	−0.18	−2.64	<0.01
Depression	Maternal IBS Status	0.4	6.24	<0.001
	Child Age	−0.05	−0.74	ns
	Child sex	0.07	1.14	ns
	Maternal Age	0.01	0.06	ns
	Maternal Ethnicity	−0.11	−1.71	ns
	Maternal Education	−0.07	−1.07	ns
	Marital status	0.1	1.49	ns
	Employment status	0.01	0.15	ns
	Number of children	−0.16	−2.55	<0.05

**Table 6:** Crosstabs for school days missed, doctor visits, and medication use.

	Only Child	Multiple Children	χ^2^	*p*
Missed School Days			8.29	<0.01
*n*	26	68		
% of sample	19.00%	33.20%		
Doctor Visits			0.02	ns
*n*	14	20		
% of sample	10.20%	9.80%		
Medication Use			0.25	ns
*n*	38	51		
% of sample	28.80%	26.30%		

**Figure A1:**
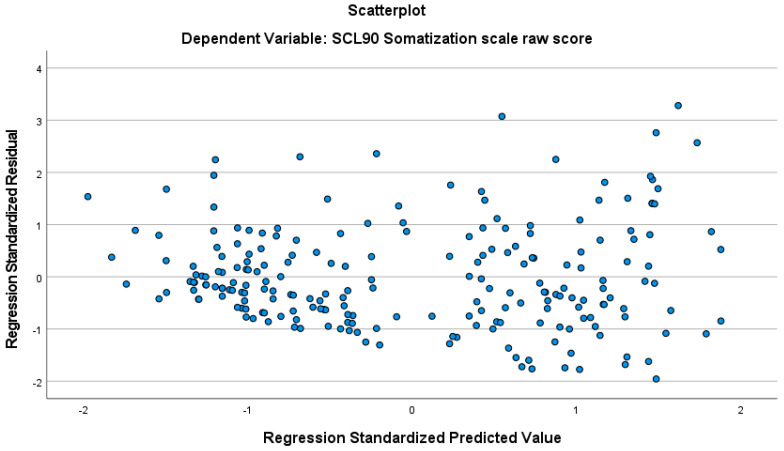
Scatterplot showing standardized residuals for SCL-90 Somatization scale.

**Figure A2:**
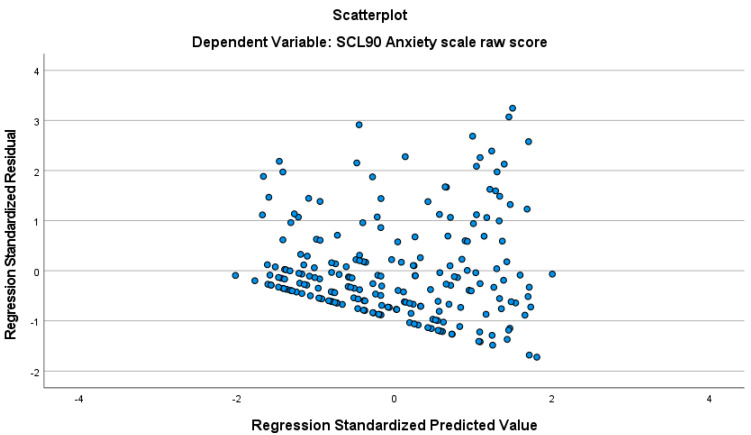
Scatterplot showing standardized residuals for SCL-90 Anxiety scale.

**Figure A3:**
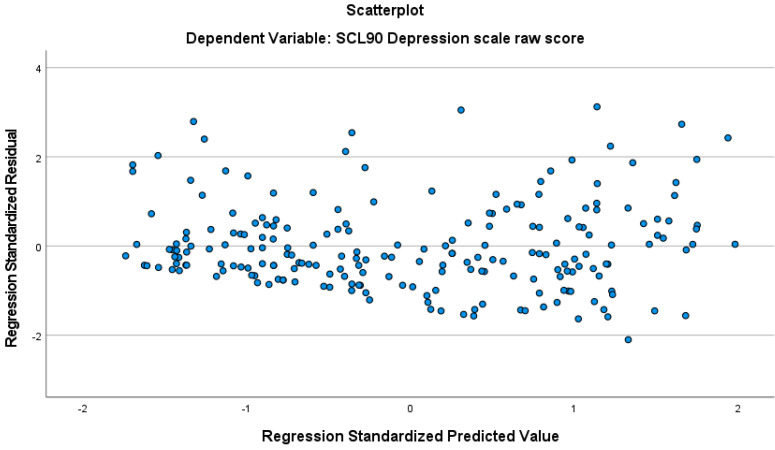
Scatterplot showing standardized residuals for SCL-90 Depression scale.

## Data Availability

The data presented in this study are available on request from the corresponding author.
